# Supplementary material for: Microdose lithium improves behavioral deficits and modulates molecular mechanisms of memory formation in female SAMP-8, a mouse model of accelerated aging
Source: PLoS One. 2024 Apr 4;19(4):e0299534. doi: 10.1371/journal.pone.0299534 (PMC10994667; doi:10.1371/journal.pone.0299534)

NMDA

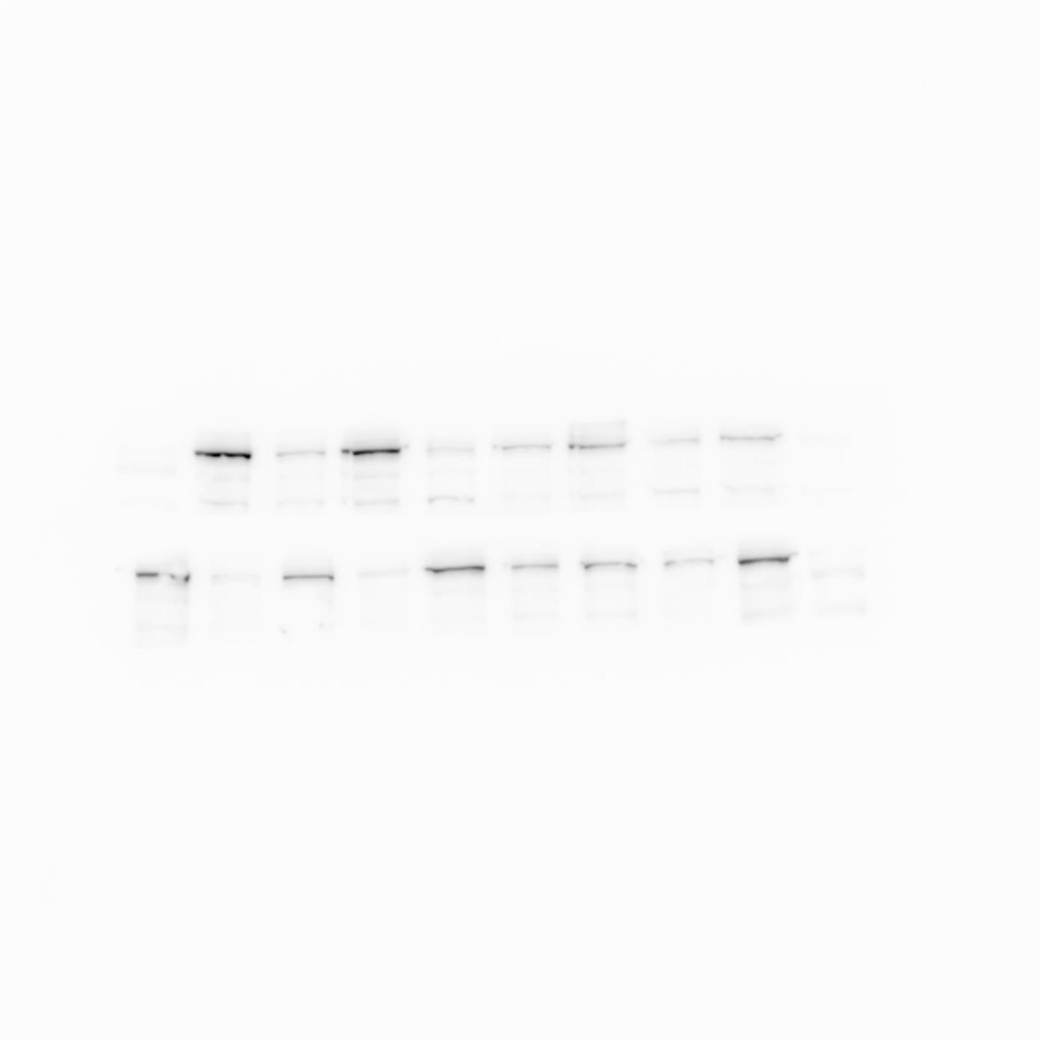

AMPA

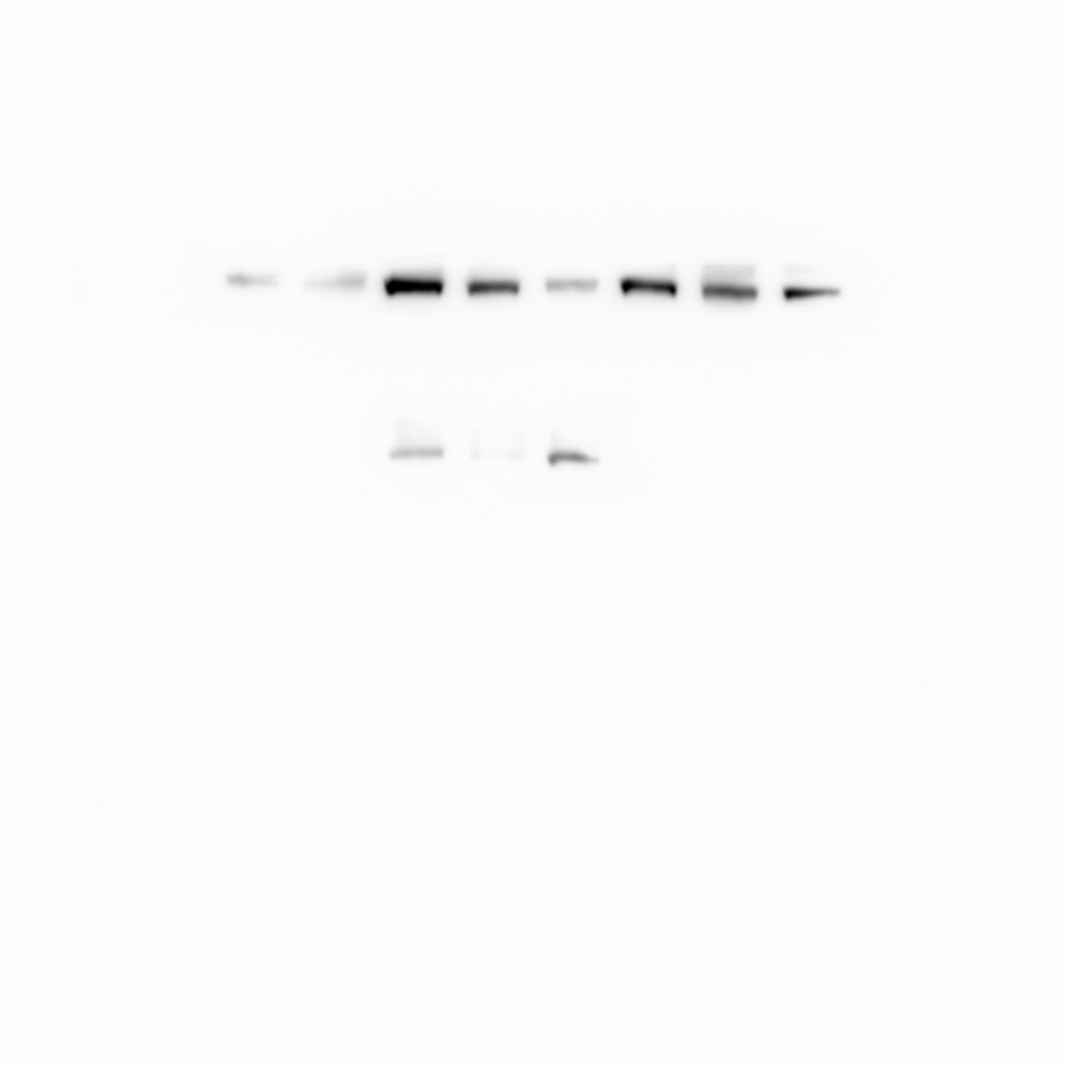

PSD95

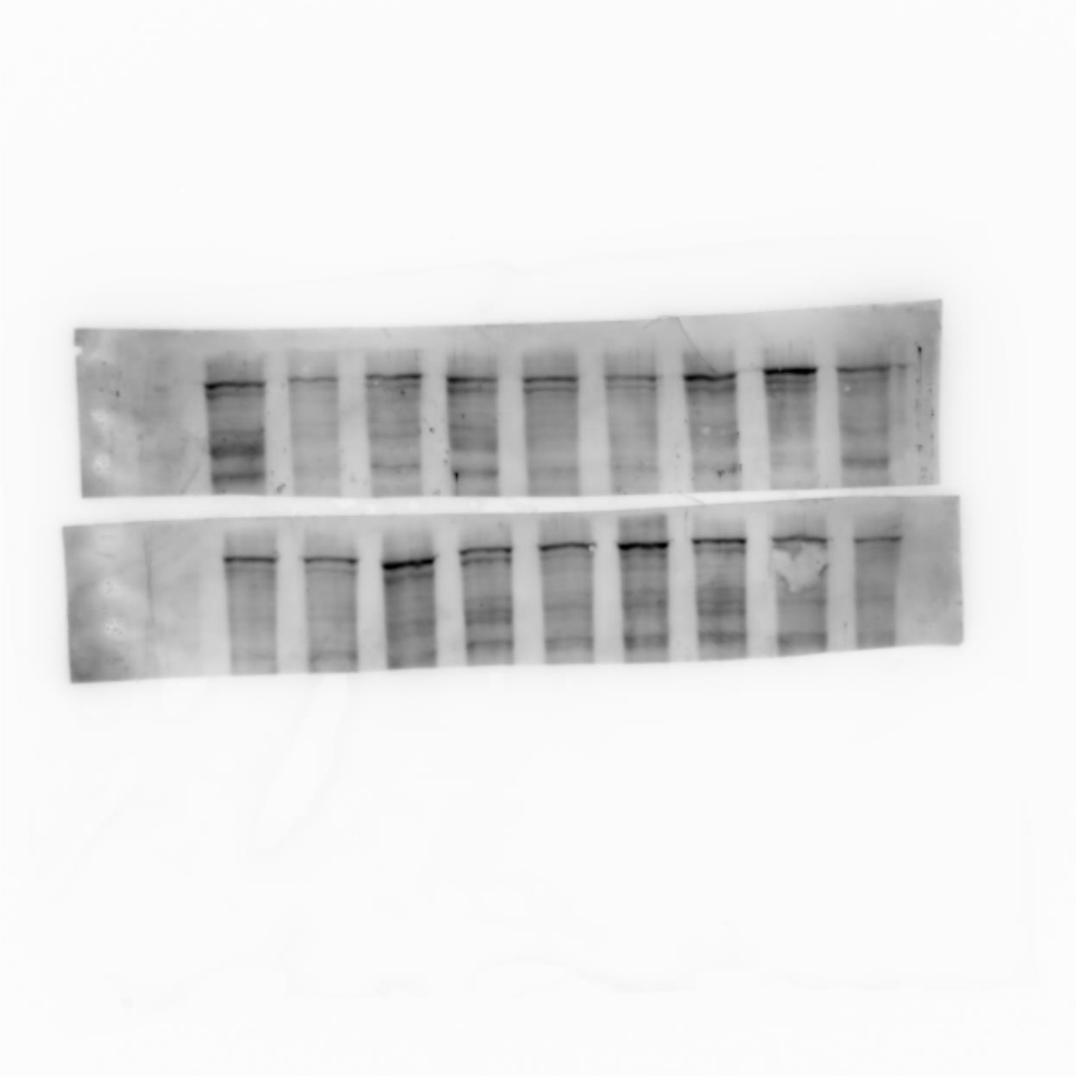

CaMK IV

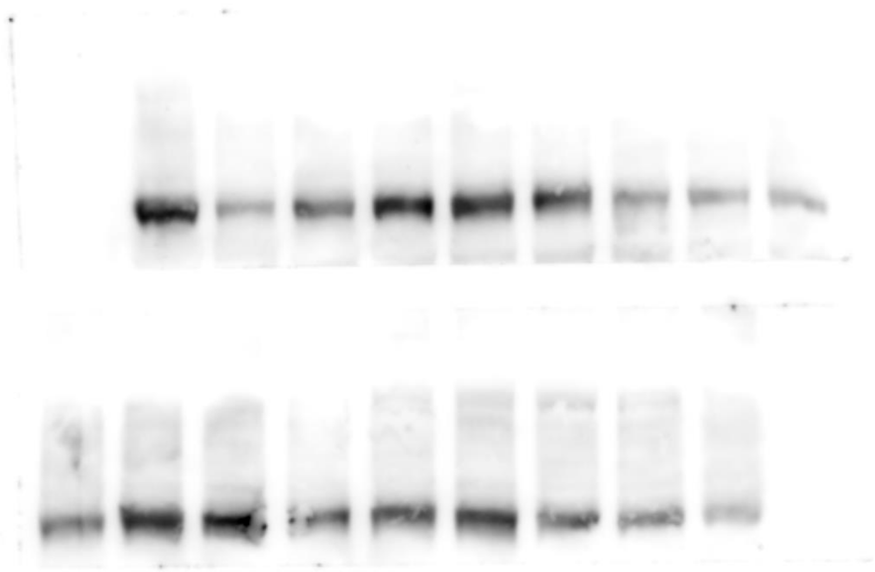

Alpha 7

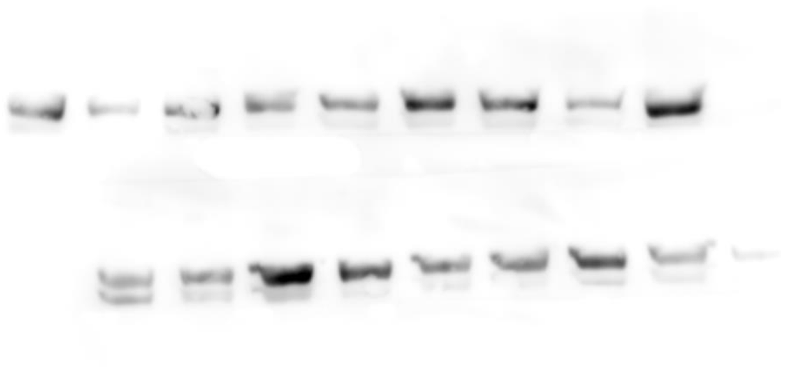

GABAa

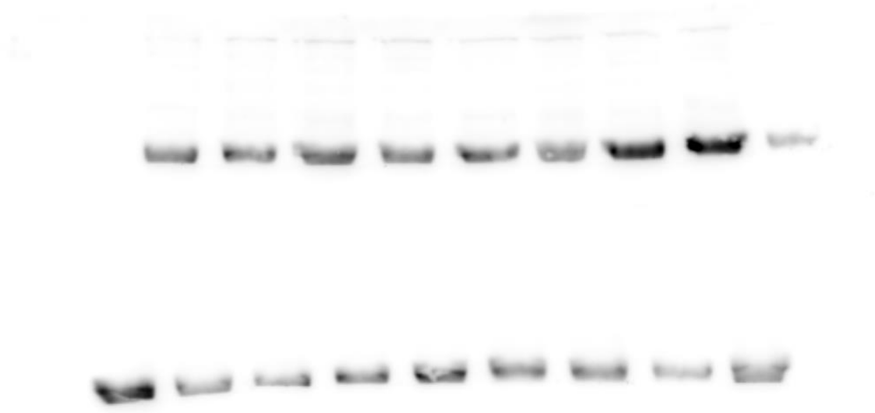

Synaptophysin

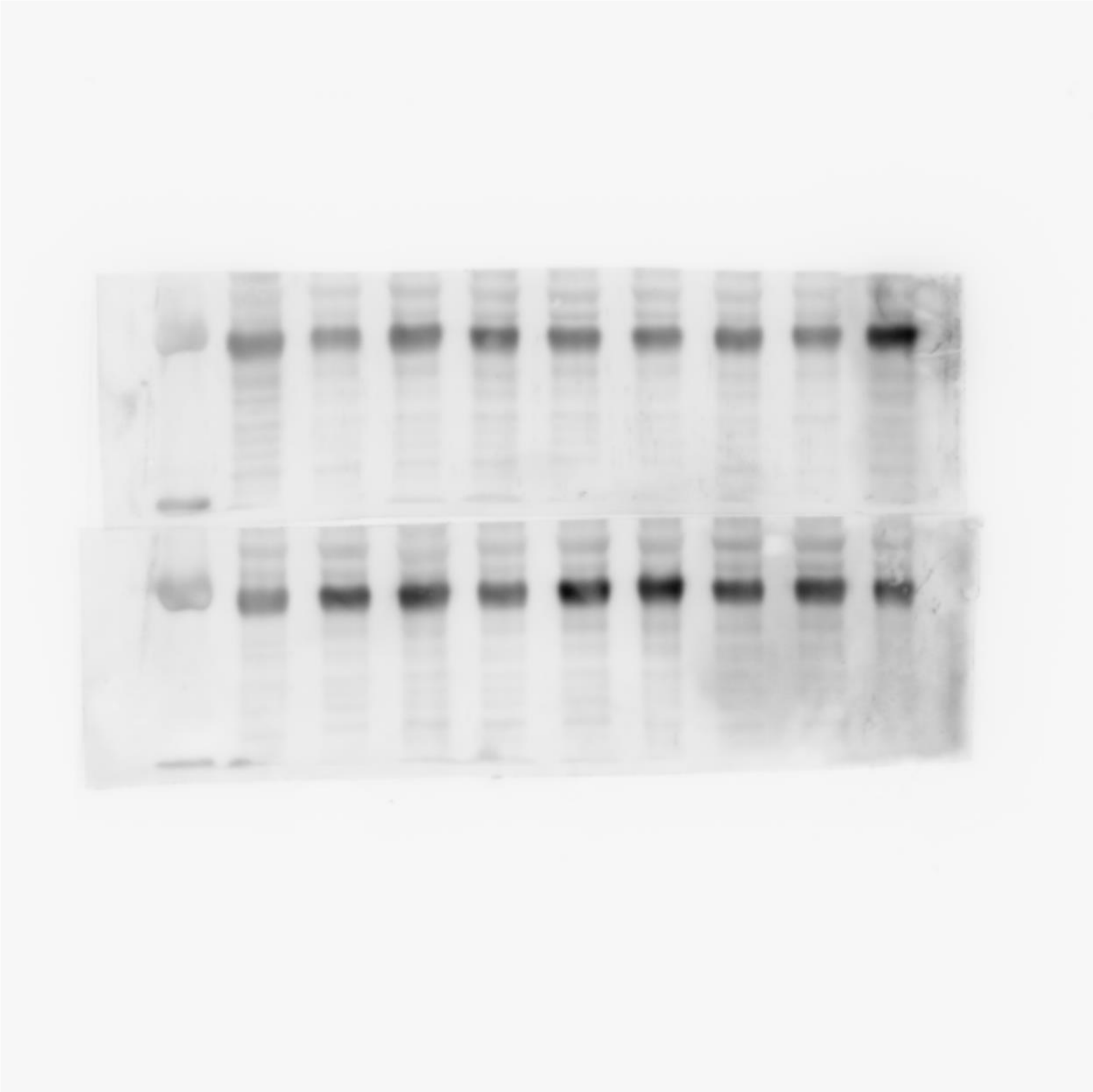

GSK3b

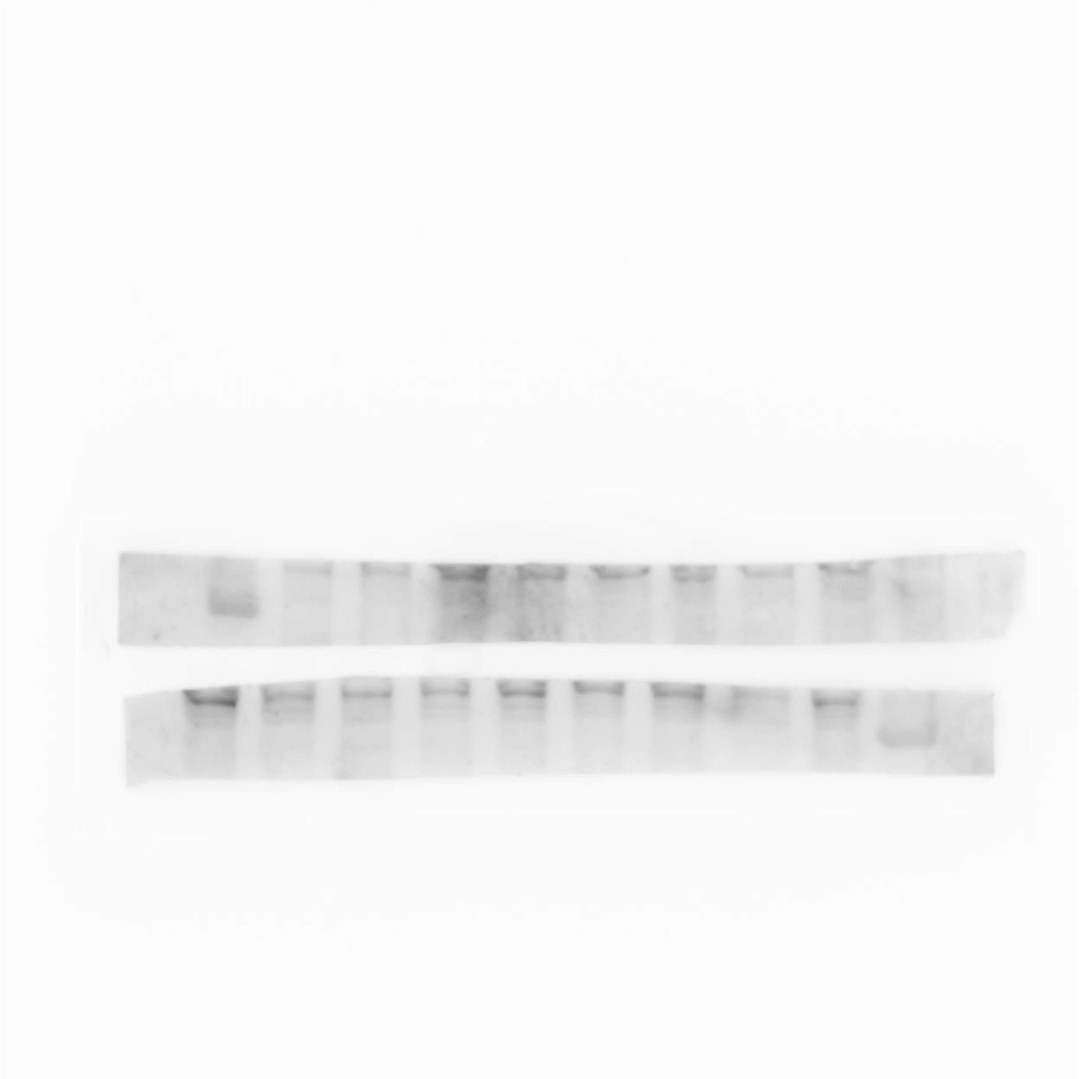

pGSK3b

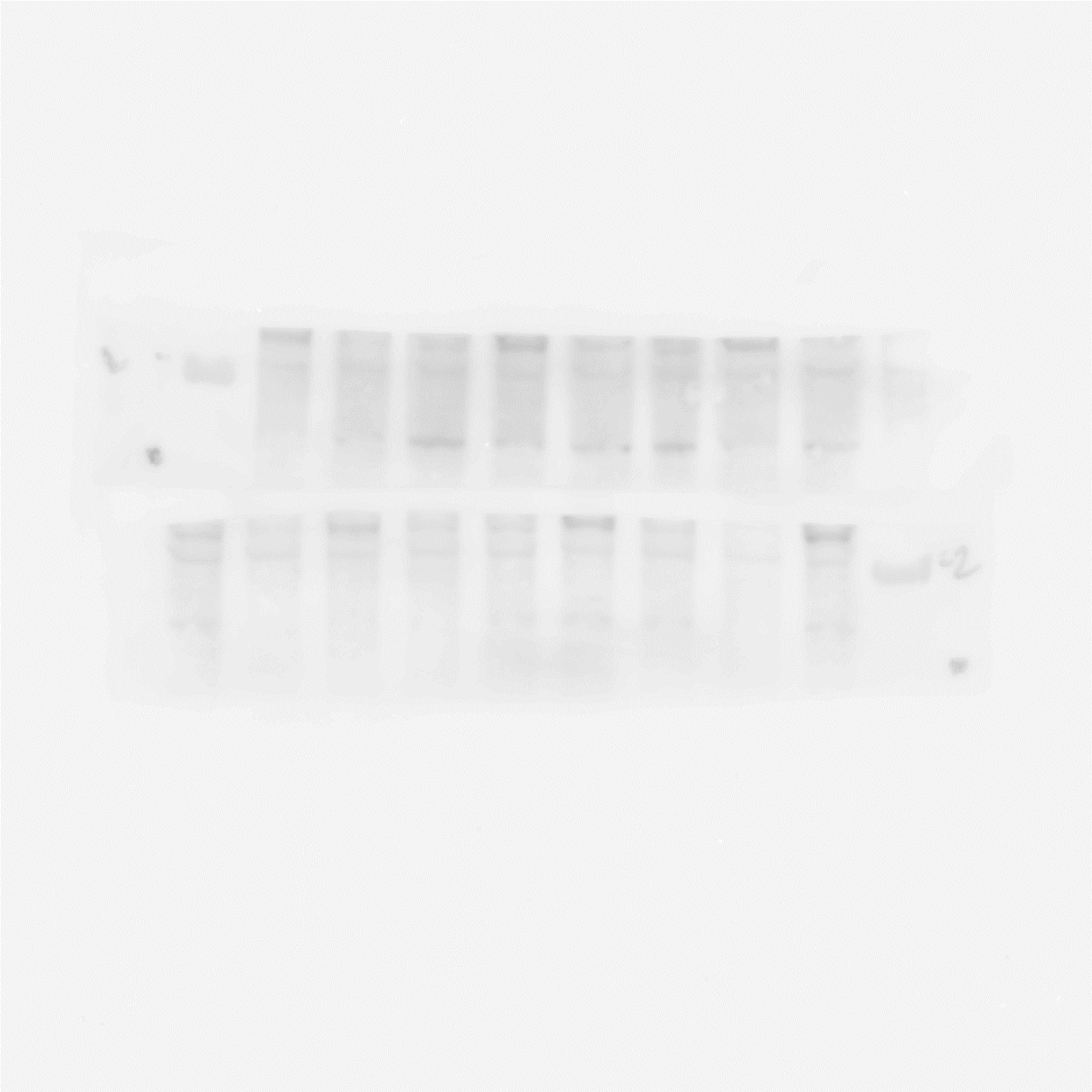

Actin (ctl of other proteins)

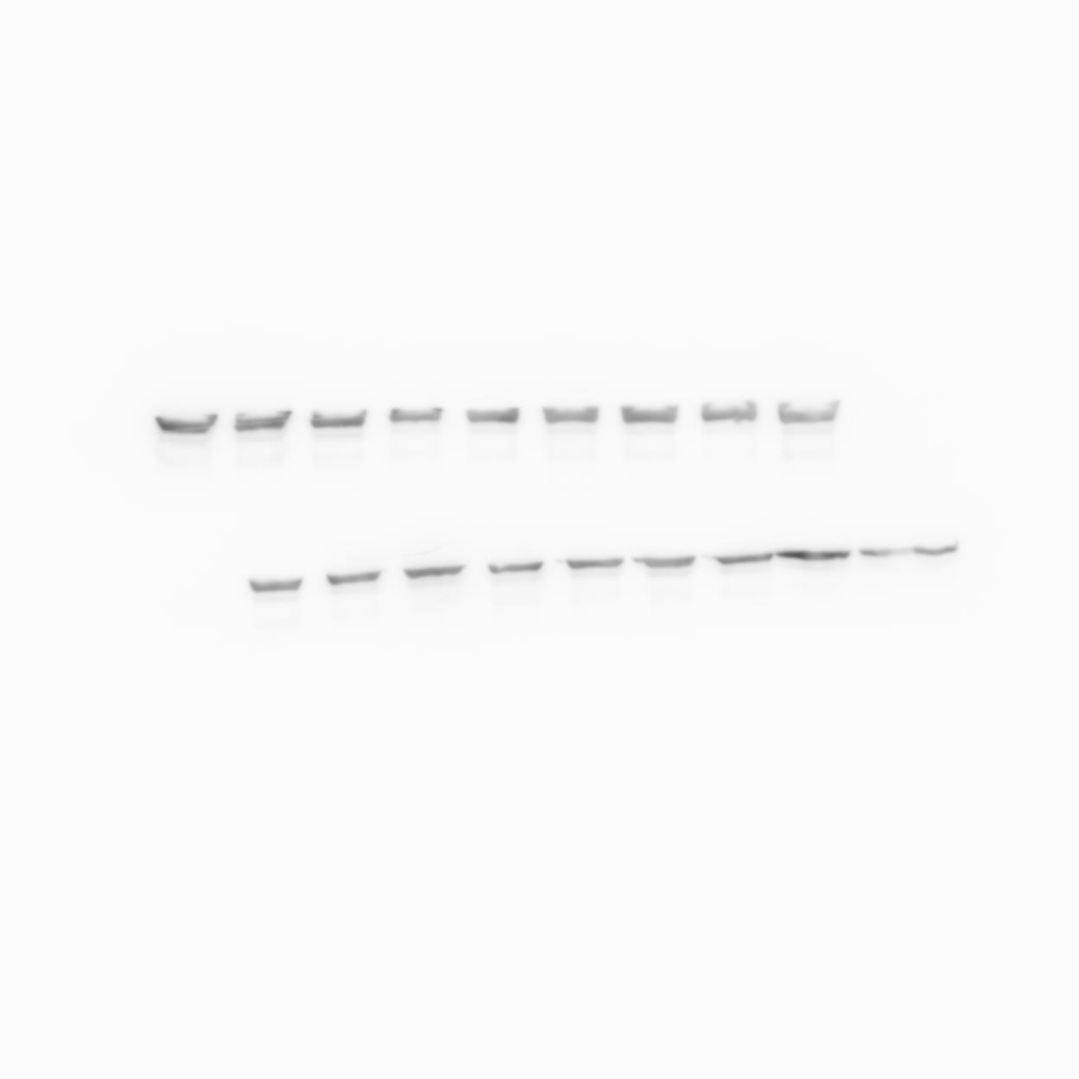

Actin (ctl CaMK)

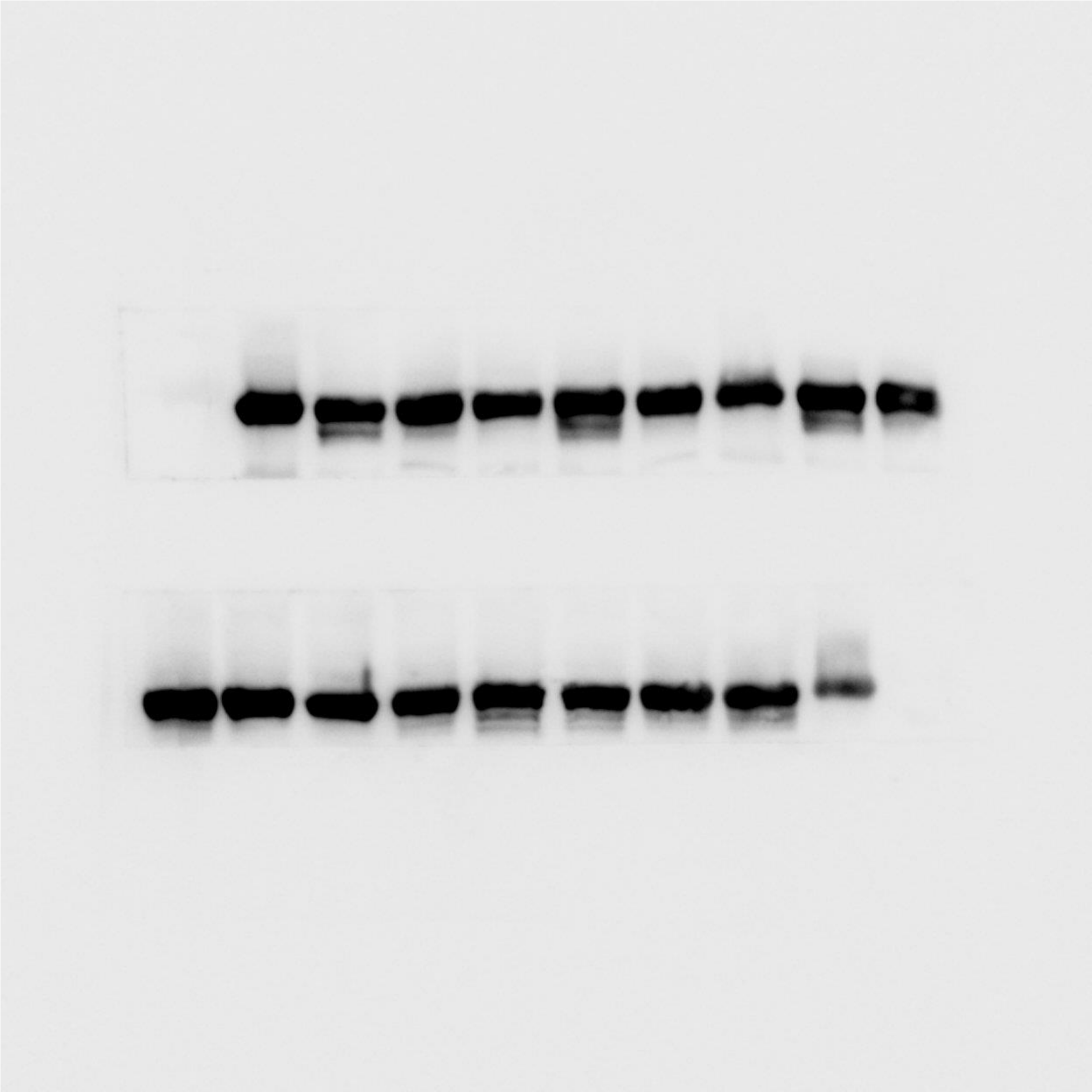

Ponceau (ctl of other proteins)

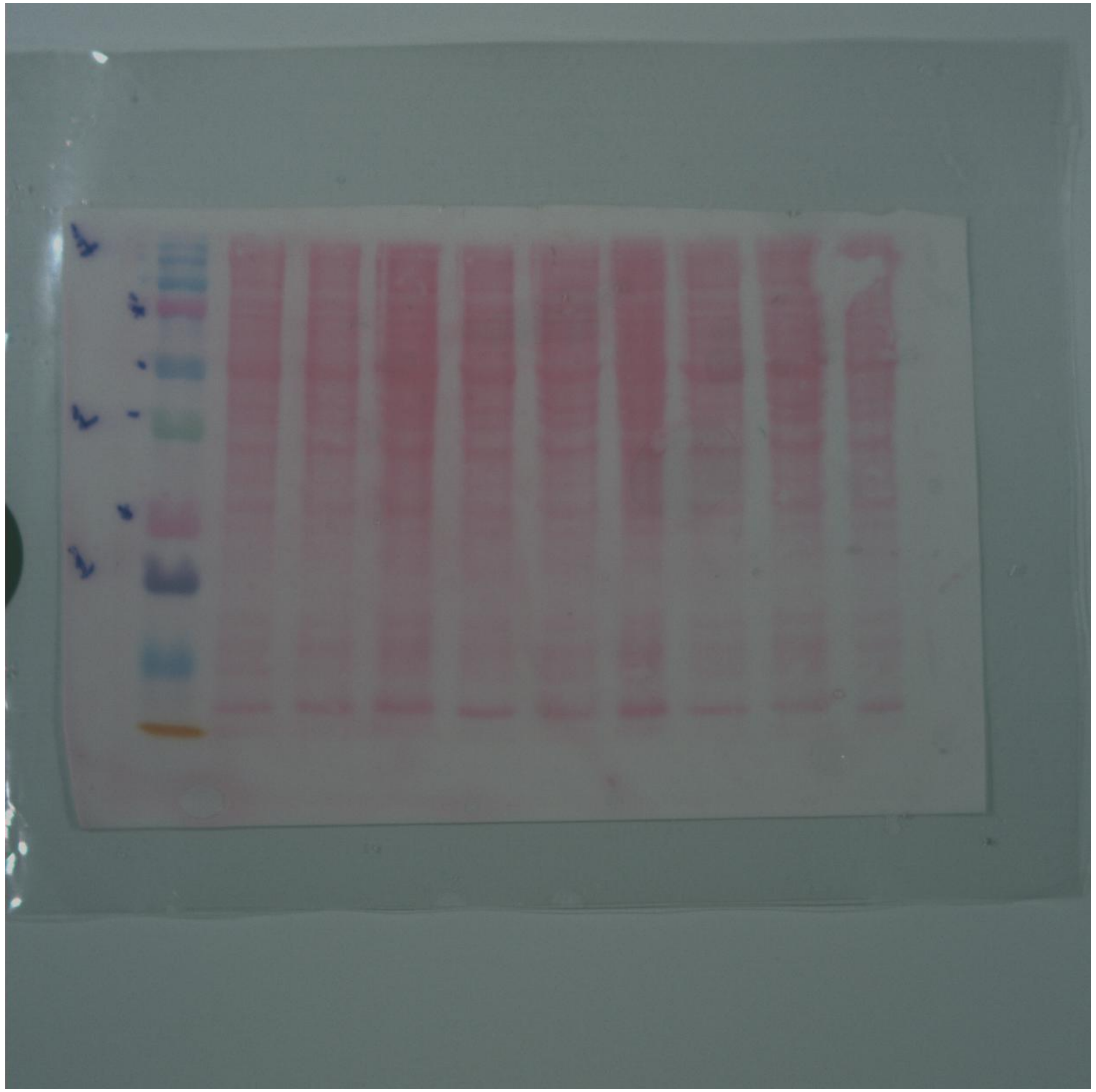

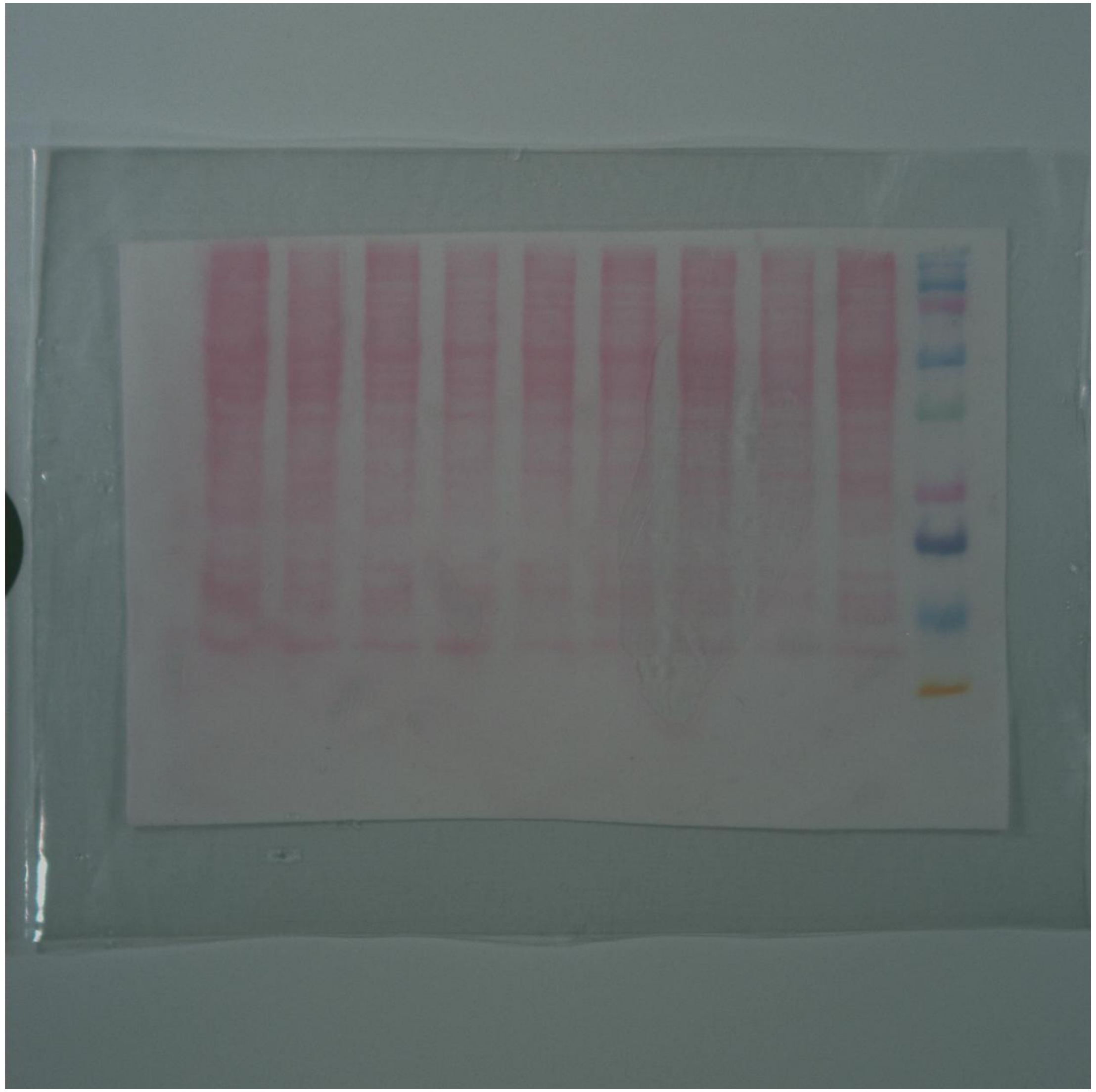

Ponceau (ctl CaMK)

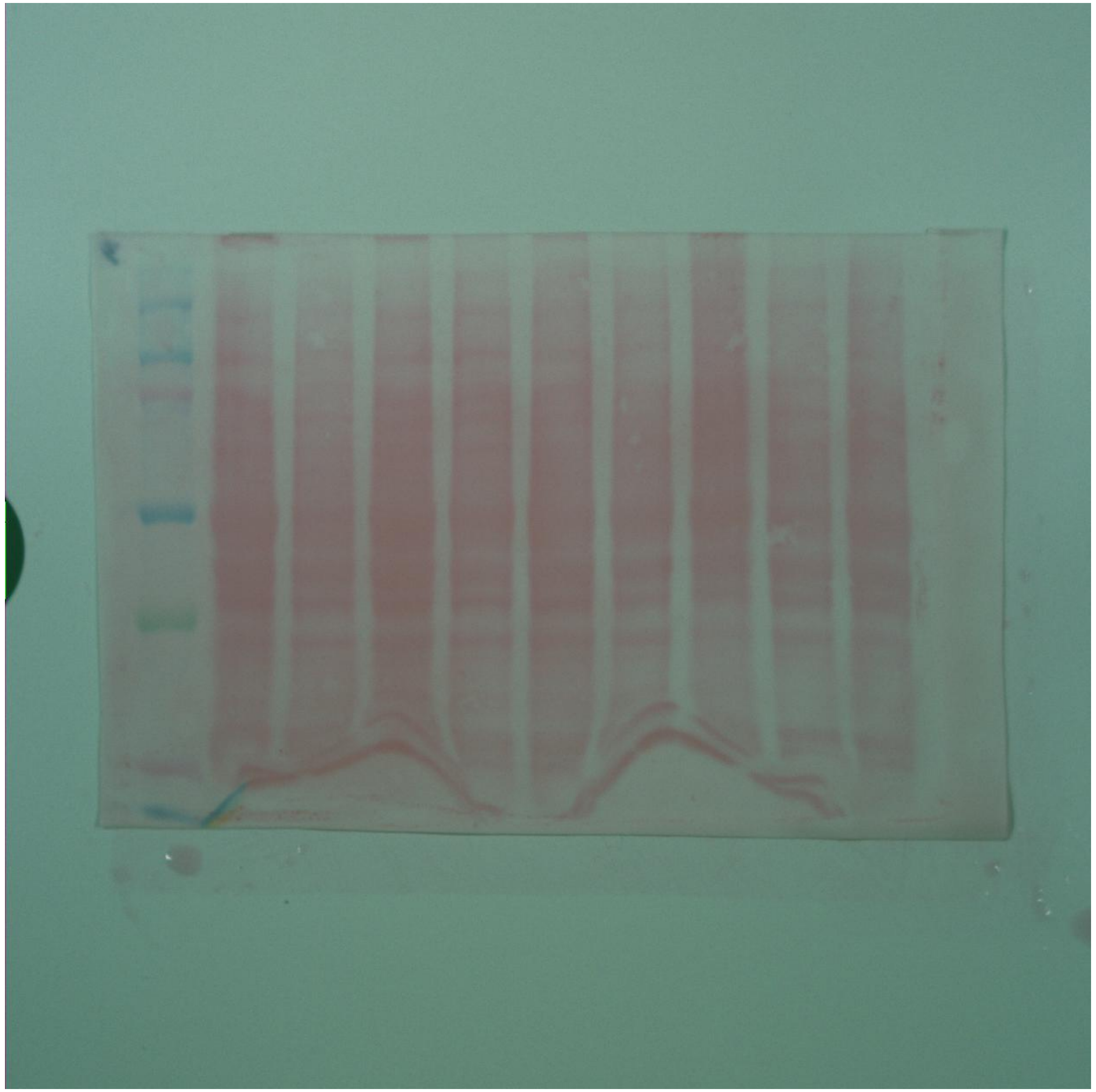

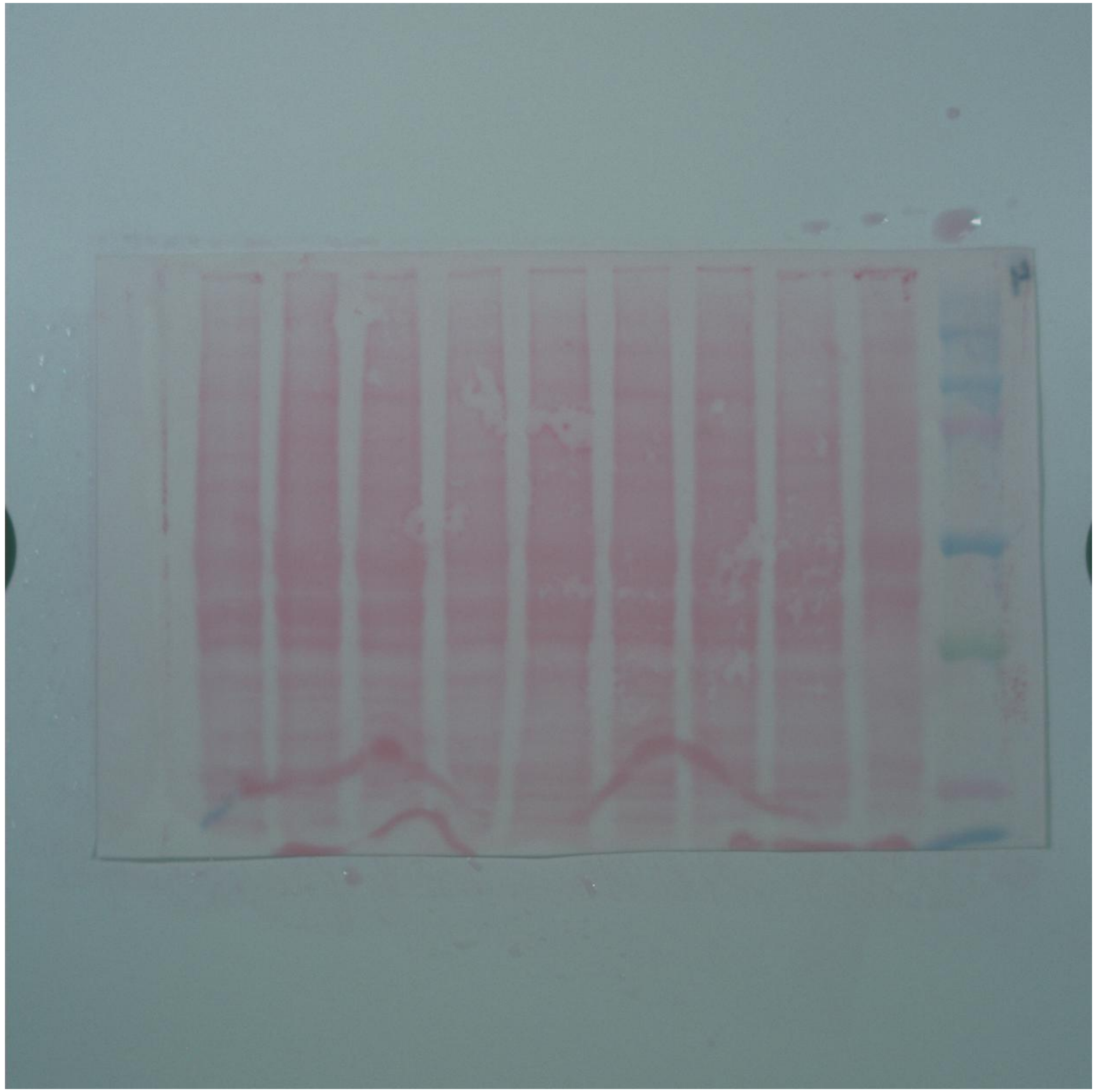

Supplement: S1 File — (PDF) [file pone.0299534.s001.pdf]
