# Supplementary material for: Microdose lithium improves behavioral deficits and modulates molecular mechanisms of memory formation in female SAMP-8, a mouse model of accelerated aging
Source: PLoS One. 2024 Apr 4;19(4):e0299534. doi: 10.1371/journal.pone.0299534 (PMC10994667; doi:10.1371/journal.pone.0299534)
Supplement: Attachment — Submitted filename: Review.pdf [file pone.0299534.s003.pdf]

Arthur Antonio Ruiz Pereira et al., in their article "Multiple mechanisms of microdose lithium protect behavioral deficits and molecular mechanisms for memory formation in SAMP-8, a mouse model of accelerated aging.," apply chronic treatment with lithium to show its effects on spatial memory, anxiety, and molecular mechanisms related to long- term memory formation during the aging process of a mouse model of accelerated aging (SAMP-8).

**Critique**:

I. **Introduction**.

1. The authors used long-term microdose lithium to treat SAMP-8 mice as a model of late-onset AD. The authors admit that the hypothesis is not novel, and lithium was suggested as an anti-AD drug by several groups. The authors should reference the papers related to the topic.
   a. Christopher Baethge (2020) stated that low-dose lithium is efficient against dementia (1)
   b. Robert Haussmann et al. (2021) suggested that lithium is a therapeutic option in Alzheimer's disease (2).
   c. Shanquan Chen et al. (2022) performed a retrospective cohort study to show the Association between lithium use and the incidence of dementia (3).
2. The novelty of the study is not presented clearly. The authors mention their clinical study with low-dose lithium. Of note, short-term treatment with lithium did not demonstrate promising results (4). However, long-term treatment protocol has been applied already. Orestes V. Forlenza et al. (2019) investigated the clinical and biological effects of long-term lithium treatment in older adults with amnestic mild cognitive impairment. They ran a long-term (24-month-long) randomised clinical trial to show the effects of lithium (5).

Moreover, a systematic review by Sivan Mauer et al. (2014) demonstrated that Lithium, in both standard and trace doses, appears to have biological benefits for dementia (6). Shinji Matsunaga et al. (2015) performed a systematic review and Meta-Analysis of several clinical trials to conclude that lithium treatment may have beneficial effects on cognitive performance in subjects with MCI and AD dementia.

Therefore, we know that micro-doses of lithium (15-300 µg) are efficient against dementia and have little side-effects in long-term protocols.

Consequently, the authors must clearly state the hypothesis and experimental goals while considering animal welfare for long-term experiments.

II. **Methods.**

1. **Treatments**. The authors must justify the dose applied and present the calculations. Unfortunately, the reference they provide does not have it.
2. **Experimental design.** The authors use only females in their study. Please clarify and justify it.
3. **Behavioral tests.** Barnes maze. The authors apply a very unusual protocol for the Barnes maze, which generally assesses spatial memory acquisition and recall. And here. The authors repeated the task when animals were 5, 8, and 10 months old to assess memory recovery. So, please define the term "memory recovery" and present a reference showing the method.

4. **Evaluation of the density of senile plaques with Thioflavin S**. The authors used a solution of thioflavin S (Sigma T1892) to stain amyloid plaques in SAMP-8 mice. The same method has been used by numerous labs in the transgenic AD animals. However, aged SAMP-8 mice, in contrast to AD mice, do not present amyloid-beta plaques detectable with thioflavin S (7). Please explain or apply another staining (IHC) for amyloid to show changes.

**III. Results.**

1. **Fig. 2.** The authors applied repeated measures ANOVA to show the effects of treatment with low-dose lithium on spatial memory, however, the presentation of the results is not correct. You cannot demonstrate statistical significance with repeated measures ANOVA by the way you did it (at the points of measurements) instead, you must analyze the entire plot and compare the curves.
2. **Fig. 8.** You are only displaying artifacts, no plaques. Look at reference #7 fig. 3.

IV. **Discussion**. There is no molecular mechanism explaining the effects of low-dose lithium. I propose a theory that could be a good fit.

1. In 1986 Adlercreutz et al. showed that Lithium lowers renal, cardiac and splenic ornithine decarboxylase activity (ODC) in mice (8).
2. In 1992 Gad M. Gilad Gad Gilad et al. demonstrated that chronic lithium treatment suppresses ornithine decarboxylase (ODC) activity in the brain (9).
3. Kan et al. (2015) convincingly demonstrated that pharmacologic disruption of the arginine utilization pathway by an inhibitor (DMFO) of arginase and ornithine decarboxylase protects the mice from AD-like pathology (10).
4. Polis et al. (2018) showed that arginase inhibition reverses cognitive decline and synaptic loss in a murine model of Alzheimer's Disease (11).

Accordingly, to enhance the quality of their work and deliver a unique and noteworthy research paper, the authors must employ additional techniques to investigate the hypothesis based on the findings of various groups about the influence of lithium salts on arginase and ODC expression levels and activities. It might be WB, IHC, or another method. I believe you have the lysates and the brains to run an experiment.

V. It is necessary to improve the quality of scientific English language. This includes correcting any errors in spelling, grammar, and punctuation to make the text clearer and more precise.

**References**:


1. Baethge, C. Low-dose lithium against dementia. Int J Bipolar Disord 8, 25 (2020). https://doi.org/10.1186/s40345-020-00188-z
2. Haussmann R, Noppes F, Brandt MD, Bauer M, Donix M. Lithium: A therapeutic option in Alzheimer's disease and its prodromal stages? Neurosci Lett. 2021 Aug 24;760:136044. doi: 10.1016/j.neulet.2021.136044. Epub 2021 Jun 10. PMID: 34119602.
3. Chen S, Underwood BR, Jones PB, Lewis JR, Cardinal RN. Association between lithium use and the incidence of dementia and its subtypes: A retrospective cohort study. PLoS Med. 2022 Mar 17;19(3):e1003941. doi: 10.1371/journal.pmed.1003941. PMID: 35298477; PMCID: PMC8929585.
4. Hampel H, Ewers M, Bürger K, Annas P, Mörtberg A, Bogstedt A, Frölich L, Schröder J, Schönknecht P, Riepe MW, Kraft I, Gasser T, Leyhe T, Möller HJ, Kurz A, Basun H. Lithium trial in Alzheimer's disease: a randomized, single-blind, placebo-controlled, multicenter 10-week study. J Clin Psychiatry. 2009 Jun;70(6):922-31. PMID: 19573486.

5. Forlenza, O., Radanovic, M., Talib, L., & Gattaz, W. (2019). Clinical and biological effects of long-term lithium treatment in older adults with amnestic mild cognitive impairment: Randomised clinical trial. The British Journal of Psychiatry, 215(5), 668-674. doi:10.1192/bjp.2019.76
6. Mauer S, Vergne D, Ghaemi SN. Standard and trace-dose lithium: a systematic review of dementia prevention and other behavioral benefits. Aust N Z J Psychiatry. 2014 Sep;48(9):809-18. doi: 10.1177/0004867414536932. Epub 2014 Jun 11. PMID: 24919696.
7. Porquet D, Andrés-Benito P, Griñán-Ferré C, Camins A, Ferrer I, Canudas AM, Del Valle J, Pallàs M. Amyloid and tau pathology of familial Alzheimer's disease APP/PS1 mouse model in a senescence phenotype background (SAMP8). Age (Dordr). 2015 Feb;37(1):9747. doi: 10.1007/s11357-015-9747-3. Epub 2015 Feb 8. PMID: 25663420; PMCID: PMC4320125.
8. Adlercreutz C, Rosengren E, Uvelius B. Lithium lowers renal, cardiac and splenic ornithine decarboxylase activity in mice. Experientia. 1986 Apr 15;42(4):409. doi: 10.1007/BF02118632. PMID: 3007201.
9. Gilad GM, Gilad VH, Wyatt RJ, Casero RA Jr. Chronic lithium treatment prevents the dexamethasone-induced increase of brain polyamine metabolizing enzymes. Life Sci. 1992;50(18):PL149-54. doi: 10.1016/0024-3205(92)90289-2. PMID: 1313939.
10. Kan MJ, Lee JE, Wilson JG, Everhart AL, Brown CM, Hoofnagle AN, Jansen M, Vitek MP, Gunn MD, Colton CA. Arginine deprivation and immune suppression in a mouse model of Alzheimer's disease. J Neurosci. 2015 Apr 15;35(15):5969-82. doi: 10.1523/JNEUROSCI.4668-14.2015. PMID: 25878270; PMCID: PMC4397598.
11. Polis B, Srikanth KD, Elliott E, Gil-Henn H, Samson AO. L-Norvaline Reverses Cognitive Decline and Synaptic Loss in a Murine Model of Alzheimer's Disease. Neurotherapeutics. 2018 Oct;15(4):1036-1054. doi: 10.1007/s13311-018-0669-5. PMID: 30288668; PMCID: PMC6277292.
