# Supplementary material for: Microdose lithium improves behavioral deficits and modulates molecular mechanisms of memory formation in female SAMP-8, a mouse model of accelerated aging
Source: PLoS One. 2024 Apr 4;19(4):e0299534. doi: 10.1371/journal.pone.0299534 (PMC10994667; doi:10.1371/journal.pone.0299534)
Supplement: Attachment — Submitted filename: Answer to Reviewers.doc [file pone.0299534.s002.doc]

# Dear Editor,

I want to thank you and the reviewers for the careful analysis of the manuscript and thank you for the opportunity to answer the questions and send a revised version of this work. We have now provided a more accurate description of the experimental procedures and analysis. We feel that the comments have helped us to improve the manuscript significantly.

As reviewers asked for significant and different changes to the figures, I hope we can answer and satisfy their questions. Also, as Reviewer 2 asked to change the data analysis, all graphs (except those related to the Barnes’ maze data) and statistical analysis were altered. Please find in the following text and the attached files a point-by-point response to the reviewers’ comments.

Best regards,

Tania Viel (in the name of the authors)

# Reviewer #1

# REVIEW REPORT FOR PLOS ONE

**Multiple mechanisms of microdose lithium protect long-term potentiation pathway and behavioral deficits in SAMP-8, a mice model of accelerated aging.**

This study provides relevant data for supporting the neuroprotective benefits of chronic treatment with lithium in the aged brain and under neurodegeneration of SAMP8 mice, a model of accelerated aging and Alzheimer's Disease. Personally, authors present excellent results, but they need to do some MAJOR CHANGES and a better discussion of them to improve the presentation of the study and the quality of it for its publication in Plos One. In particular, I suggest the following improvements:

1. I do not feel qualified for extensive English editing, but I think that authors should modify “protects” in the title for “protect”. They may be send the article for English editing and avoids these mistakes.

**Answer: Thank you for the observation. The correction was made, and a complete revision of the English writing was performed.**

2. Line 12. Explain abbreviation of GABAa in the abstract.

**Answer: It was explained in the abstract (line 14).**

3. Line 14. Explain abbreviation of GSK-3β in the abstract.

**Answer: It was explained in the abstract (line 16).**

4. Introduction must be modified and rewrited. Authors should be focus on two main points in the introduction: I) to contribute to data regarding to SAMP8 mice as model of accelerated senescence, and explain their neurodegenerative processes and cell damage during their brain aging and; II) to contribute to data regarding the benefits of lithium for the brain pathologies as well as some mechanisms involved on its neuroprotection. In this way, paragraph from line 33 to line 39 must be deleted. Data in the paragraph from line 40 to line 53 may be included in the discussion, since it is better to discuss these previous data from the authors in the section of discussion instead to in the introduction.

**Answer: The introduction was rewritten and the focus was the benefits of low-dose lithium for psychiatric and neurologic disorders, together with an explanation of the term "microdose" used in the title and all the papers we published until now (pages 3 and 4).**

5. Lines 44. Explain abbreviation of BDNF.

**Answer: As this paragraph was included in the Discussion, the term was explained in this part of the manuscript (line 546).**

6. Information provided in Animals, from line 66 to line 70 regarding SAMP8 mice should be better included in the introduction. This section is the best place to explain details regarding the experimental design, including final number of animals and the figure 1. In this way, data provided from line 165 to line 178 should be better presented here. Also, I think that is not necessary to provide information about those dead animals. Authors must be provide the final number of SAMR1 animals, SAMP8 animal and SAMP8 treated with lithium that they finally used to obtain the results of this study.

**Answer: Information about SAMP-8 mice was included in the Introduction (lines 80 to 83) and we took out the information about the dead animals, living only the final number of animals in each group, as suggested.**

7. Could authors explain why have only been working with females animals?

**Answer: Many studies show that the prevalence of Alzheimer's disease in women is greater than in men (for examples, please verify Niu et al., 2017; Beam et al., 2018), and the authors attribute this phenomenon to "women’s survival to older ages compared to men". In fact, we published another work with male SAMP-8 mice, and some molecular events (protein densities) were not the same as in female mice. In this way, we performed the present work separately from the other one with male mice (Malerba et al., 2021). This discussion was added to the text (Discussion, lines 505 to 512).**

8. Authors must explain the final doses of lithium daily ingested by animals and then also provide ingested volume monitored weekly (or do not comment this last point). In this paragraph they must also specify the time of treatment with lithium. Did SAMR1 and SAMP8 control animals receive some type of vehicle? May be just water? Please, specify also this point.

**Answer: More details about the final lithium dose and treatments were provided and the information about the ingested volume was suppressed (lines 51 to 54).**

9. In biochemical and histological analysis, authors should provide information about using some type of cryoprotective agent. They have done immunofluorescence analysis on frozen brain tissue, which was processed by cryostat. Please, specify this information also here.

**Answer: As described in the Methods Section - Biochemical and histological analysis, after behavioral tests, mice were anesthetized with 4% isoflurane, and the brain was collected and immediately frozen in dimethylbutane at -50ºC and stored at -80ºC until used. No cryoprotective agent was used in this method since samples were still frozen until use.**

10. Abbreviations of antibodies names included in the paragraph from line 131 to line 134 must be provided (e.g., PSD95, NMDA-R2B, AMPA, etc.). Authors should provide briefly information regarding their role on neuroplasticity and/or long-term memory.

**Answer: The antibodies' names were included together with a brief explanation of their role in neuroplasticity and neuroprotection (lines 160 - 184).**

11. Why authors did use brain sections of 20 μm whether cryostat permits do much thinner sections?

**Answer: In our experiments, each mouse brain is prepared in a cryostat to obtain 5 to 6 sets of brain samples that cover all the brain, from rostral to caudal areas. In this way, all brains are prepared for immunohistochemistry and radioautography analysis. According to our experience of more than 15 years, 20 µm brain sections permit us to work with both techniques (please see some examples in the following works, published in journals with selective editorial policy: Viel TA et al. Neurobiol Aging. 2008; Viel TA et al. Curr Alzheimer Res. 2012;**  **Baraldi T et al. Exp Gerontol. 2013; Telles-Longui et al., Br J Pharmacol. 2019; Malerba et al. Front in Aging Neurosci. 2021).**

12. Analysis of density of senile plaques was done by using thioflavin S. This is also inmunofluorescence, so that authors must provide type of inverted microscope used (confocal or fluorescence microscope?). Specify this information here. Also specify the thickness of the brain sections.

**Answer: Thioflavin S staining is not an immunofluorescence method since no antibody is used, but it is a naturally fluorescent homogenous mixture of compounds that results from the methylation of dehydrothiotoluidine with sulfonic acid. In the Methods Section - Evaluation of the density of senile plaques, the type of microscope and the software used are described. As indicated in the text, it was used an inverted fluorescence microscope (Leica, DMi8) and the LasX software (Leica Microsystems). The word “fluorescence” was added in the text. It was used 20 µm brain slices, and this information was added to the text (line 219).**

13. Move information provided in the paragraph from line 165 to line 178 to the beginning of the methods section, as comment above.

**Answer: In item 6 of the reviewer's comments, the reviewer asked to move the paragraph from lines 165 to 178 of the Introduction (lines 80 to 83).**

14. Line 185. Change “immunohistochemical” for “immunofluorescence”.

**Answer: We changed “immunohistochemical” for tissue analysis as thioflavin S was not an immunofluorescence protocol. Thank you for the observation. The change was made (line 251).**

15. In results, due to that some of animal died, the number of animals is not the same for each experiment done. For instance, results about memory were done by using 11 samr1 mice and 24 smp8 mice at 2-months of age. However, data obtained at 8 and 10 months of age were obtained by using a lesser number of animals (7-9). Given these important differences, authors must always indicate the number of animal’s samr1, samp8 and samp8+lithium that have used for each experiment and result. This place may be better to explain what happens with those dead animals, instead to in the methods section.

**Answer: This is a very nice perception, and we thank the reviewer for the suggestion. The number of animals in each result was added to the figure's legends.**

16. In the figures 2A-D, authors should clearly indicate which data is from samr1 mice or those are from samp8 mice for a better understanding of the graphs.

**Answer: The legend was added in graph 2D.**

17. Again, in line 234 indicate number of samr1, samp8 and samp8+lithium mice were used form obtained the data.

**Answer: The number of animals in each group was added to the Figure legend.**

18. In the figure 3, indicate clearly which data is from samr1 mice or those are from samp8 mice for a better understanding of the graphs. Remove the individual points from the histograms in order to better visualize the SEM in the graphs.

**Answer: A legend was added to clarify which data are from SAMR1 and which are from SAMP8. All the graphs were changed according to this reviewer and reviewer 2 suggestions.**

19. Regarding molecular markers related to memory and neuroplasticity, authors must always indicate in the text: a) percentage of decrease in samp8 versus samr1; b) percentage of increase in samp8 + lithium versus samp8 (if there was); and c) percentage of increase in samp8 + lithium versus samr1 (if there was).

**Answer: The percentages were added to the text (lines 332 to 381)**

20. In the figures 4 and 5, indicate clearly which data is from samr1 mice or those are from samp8 mice for a better understanding of the graphs. Remove the individual points from the histograms in order to better visualize the SEM in the graphs.

**Answer: The alterations were made.**

21. Figures 5-9 must be reorganized in order to show images from microscope together with their specific quantification. In this way, the new order would be: a) Figure 6 together with figure 5 A and 5D; b) Figure 7 together with Figure 5B and 5E; c) Figure 8 together with figure 5C and 5F; and d) Figure 9 together with figure 5G.

**Answer: Those figures were changed and organized as suggested (Figures 5 to 8).**

22. In figure 5C, Why are there a “a” in the statistical differences instead to an asterisk?

**Answer: We use to put an “a” or an “b” when the significance is greater than P < 0.001. This is a matter of style, as we used this labeling in other papers already published. But in this version, we’ll follow the suggestion and use only asterisks (Figures 3 and 4).**

23. In the figure 9, in which regions of the hippocampus was done the analysis of senile plaques? Only in the dentate gyrus? Please, indicate in the figure 9 as well as in the text (results and methods).

**Answer: As described in the Method’s section (line 226), the analysis was done in CA1, CA3, and GrDG hippocampal areas. This information was added to the legend of Figure 9 (now named Figure 8).**

24. I would not make sections in the discussion. Discussion is the worst part of this study. Authors have not been a good discussion of their results, and this section is really difficult to understand. Therefore, discussion must be rewrite. Also, an extensive English editing is necessary.

**Answer: The Discussion was thoroughly revised.**

25. Data obtained previously from the authors must be appropriately detailed at the beginning of the discussion, with their specific references. Some affirmations about their previous data have not reference; for instance, lines 373-375, lines 380-382, lines 444-446, lines 448-449.

**Answer: Thank you for the observation. The references were added (lines 493, 501). The text in lines 444-446 refers to other mentioned papers. But we agree that the text is confusing, and we have already changed. A complement to this reasoning was added (lines 604-608). Information given in lines 448-449 is not our authorship but common knowledge. That is why we did not put a reference at this point, but we quote seven works along the following sentence.**

26. Line 396. Data of NMDA receptors in male SAMP8 mice…please indicate age of these mice, brain zone studied, etc. Information must be given detailed.

**Answer: The information was added to the text (lines 520-521).**

27. Lines 398-399. Relationship between female hormones and NMDA receptors must be discussed in detailed.

**Answer: We thank the referee for the suggestion, but the authors think that the Discussion is already long and the influence of female hormones in memory formation is not the focus of this paper. In this way, we agreed that the information and the three references provided are sufficient for the objective of discussing this point.**

28. Line 410. Results of senile plaques in previous studies from the authors also in samp8 brain samples must be detailed (age, treatment, brain region, etc.)

**Answer: This information was added to the text (line 535).**

29. Lines 418-426. Nothing is understood in this paragraph.

**Answer: The paragraph was rewritten (lines 547-548).**

30. Line 459. GSK-3β abbreviation must give before, already in methods.

**Answer: Information was taken from this part and included in the methods section (line 182).**

31. Discussion regarding proteases related to the formation of senile plaques, roles of the NF-kβ signaling in neurodegeneration, and other toxic protein aggregated in the brain of samp8 must be better discussed. Authors should revise previous data regarding these points that were already studied in the brain of SAMP8 mice, and even it was already observed neuroprotective effects of treatments with lithium in these mice. Some interesting studies are suggested as following:

- [Favorable effects of a prolonged treatment with melatonin on the level of oxidative damage and neurodegeneration in senescence-accelerated mice.](https://pubmed.ncbi.nlm.nih.gov/18410310/) Pineal Res. 2008 Oct;45(3):302-11.
- [Melatonin alters cell death processes in response to age-related oxidative stress in the brain of senescence-accelerated mice.](https://pubmed.ncbi.nlm.nih.gov/19090913/) J Pineal Res. 2009 Jan;46(1):106-14.
- [Lithium treatment decreases activities of tau kinases in a murine model of senescence.](https://pubmed.ncbi.nlm.nih.gov/18520779/) J Neuropathol Exp Neurol. 67(6):612-23.
- [Chronic administration of melatonin reduces cerebral injury biomarkers in SAMP8.](https://pubmed.ncbi.nlm.nih.gov/17439556/) J Pineal Res. 2007 Apr;42(4):394-402.

**Answer: We discussed the reviewer's suggestion and we think that there is no reason to add a discussion related to melatonin treatment or oxidative stress in the present work, even though the authors used the same animal model that we used. The 3^rd^ suggested paper used a different lithium concentration and did not analyze the proteins related to the memory formation pathway. In this way, we respect the suggestion, but we decided not to include these points in the Discussion.**

32. Results obtained from the immunofluorescence analysis are not discussed appropriately. Specially, those related with neurodegeneration (NeuN) and neuroinflammation (GFAP).

**Answer: The discussion was added to the text (lines 536-545).**

33. Data provided and discussed in the figure 10 is not supported by the results obtained by the authors in the present study. For instance, authors have not measured BDNF levels in females SAmr1 and samp8 mice. Neuroinflammation is not demonstrated in this case since data provided from GFAP analysis did not show differences at any case. Also, inflammatory receptors and mediators have not been studied here. Cell proliferation was also not evaluated in the present work. Therefore, conclusions must be reconsidered appropriately.

**Answer: We intended to summarize the effects obtained with microdose lithium in many works that the group have already published in the last years and that are cited in the manuscript. It was a mistake not to mention these works again. In this way, we included the references in that paragraph. The authors agree that Figure 10 is essential to highlight that lithium does not have a single neuroprotective mechanism, but several ones that contribute to its pharmacological properties. The references were added to the Conclusions (lines 620-624).**

34. Line 503. Correct “Aknowledgements” by “Acknowledgements”.

**Answer: Thank you for the observation.**

# Reviewer #2

The authors performed a longitudinal assessment of SAMP8 mice, to study the behavioral and molecular effects of chronic lithium microdoses. The hypothesis and rationale are solid, and the results provide possible mechanisms associated with a type of treatment which has been also tested in the clinic.

Despite the general interest and relevance for the field, the authors should address a number of points.

1)Title: No mention to LTP and mechanisms should be made as the paper do not show any Ephys data and the findings are correlative. Would rate talk about neuro-protective effects of lithium in the SAMP8 model.

**Answer: The authors would like to thank the reviewer for the observation and declare that we agree that the correlation with LTP was speculative. The term LTP was replaced for the formation of long-term memory wherever it appeared.**

2) Introduction:

Line 3: some therapies that alleviate symptoms do exist. Please re-formulate the sentence. Do you mean disease modifying?

**Answer: We agree that some therapies that alleviate symptoms exist, but there is no treatment for the disease, as we state in line 3 (“there is no pharmacological therapy to treat the symptoms). We changed the sentence to make it clearer (lines 3 and 4).**

Authors should pay much more attention in describing/discussing the background of their search in an appropriate way. eg., line 36-37: "physical activity, high education level and better nutrition" are for sure associated with cognitive research but should not be presented as active therapies as in the text.

**Answer: The sentence was removed, and the text was modified according to this suggestion and that of Reviewer 1.**

In section starting at line 40, authors should explain what is meant as microdose. How is that defined? Relative to what doses for which other indication?

**Answer: The explanation for the term used was added (lines 52-54).**

3) Methods:

Why only female mice? Please specify.

**Answer: An explanation for that was added in lines 505 - 508.**

What would be a non-micro dose of lithium?

**Answer: This was already answered, and the explanation is in lines 46-54.**

The ingested volumes of water should be placed in a table as supplementary material (eg., as monthly average in the two groups separately and expressed relative the body weight).

**Answer: As we saw no difference in the ingested volumes registered in previous works, we did not control the water consumed in the present work. The information given (“The ingested volume was monitored weekly (unpublished data)”) was removed from the text.**

Line 116: was the hippocampus dissected before or after freezing of tissue?

**Answer: As described in lines 110-111, “After behavioral tests, mice were anesthetized with 4% isoflurane, and the brain and blood were collected and immediately frozen in dimethylbutane at -50ºC and stored at -80ºC until used.” So, the hippocampus was dissected after freezing.**

Line 119: should "Basileia" rather be "Basel"?

**Answer: We thank the reviewer for the observation. The correction was made (lines 158 and 159).**

Line 142: were section post fixed in PFA before freezing and sectioning?

**Answer: No, fixation was done directly on the slides (with 100% acetone for 5 min) just before performing immunofluorescence. This information, together with other missing details, was added to the text (lines 196-199).**

Line146: please provide information on the buffer composition (PBST as below?) and blocking agent used for staining.

**Answer: We did not understand what information is missing, as in line 146, there is no mention of PBST. PBST is explained in lines 156-157: “PBS containing 0.1% Triton X-100 (PBST)”**.

Line 161: Please specify what GrDG stands for (granule layer of the dentate gyrus?)

**Answer: GrDG stands for the granular layer of the dentate gyrus. The information was added to the text (line 226).**

The experimental design should be presented right after the information on the **treatment**.

**Answer: The authors agreed that it is clearer to describe the Experimental Design after the treatments (line 118).**

Additional details on analyses if staining should be given: software used/quantification and normalization steps/number of slides per animal and per staining
**Answer: The following information was added in the Methods Section -** **Evaluation of neurons and astrocytes densities in the hippocampus (lines 207-216):**

**Images of the hippocampal regions GD, CA1, and CA3 were acquired using the Leica DMi8 inverted microscope and analyzed in the LasX software (Leica Microsystems). The filter for labelings made with NeuN was GFP with excitation at 488 nm and emission at 510 nm. The filter used for GFAP had excitation at 561 nm and emission at 594 nm. To visualize the cell nucleus, it was used a filter for DAPI with 345 nm excitation and 460 nm emission.**

**Images of six sections of each animal’s brain were acquired with a 10x objective lens between -1.70 mm to 2.46 mm, starting from bregma (PAXINOS; FRANKLIN, 2008). The brightness detection range was defined by the operator for each slice analyzed to avoid false positives and false negatives.**

4) Results

Given that the three groups (SAMR1, SAMP8 H2O, SAMP8 Li) were run in parallel in the same experiment, data from the three group should be analyzed and presented together. For all readouts, include all three groups in the same ANOVA and in Figures present the three group together (i.e., without duplicating the SAMP8 H2O group)

**Answer: All graphs were changed.**

In figure 4 and 5 I would suggest adding the line for the X axis

**Answer: Figures 4 and 5 were changed entirely as per the suggestion of Reviewer 1.**

Figure 6-9: the PDF gives images of bad quality ("pixelled"), even without zooming in. Images of better resolution should be given in the final version.

**Answer: The resolution of the figures is in accordance with the Journal’s rules. The low quality of the image is due to the conversion to PDF. The original images are of high quality.**

In Figure 9, given the high background, please indicate with arrows what was considered as signal. Describe how it was differentiated from background in Methods.
**Answer: Arrows indicating the signal were added. Signal was differentiated from the background by visualization in higher magnification and by the operator's experience. This information was added to the Methods Section - Evaluation of the density of senile plaques (lines 223-225).**

5) Conclusion:

Line 372: some therapies that alleviate symptoms do exist. Please re-formulate the sentence. Do you mean disease modifying?

**Answer: As answered before, that are some therapies that may alleviate the symptoms, but there is no effective treatment to cure the disease. The sentence was changed for a better understanding of the argument (lines 490-491).**

Authors should refrain from referring to much to LTP in the Conclusion (and also in the Title), as they do not present any Ephsy data. The data mostly points towards general neuroprotection of synaptic function, but no direct link specific synaptic mechanisms should be made as they are not directly assessed.

**Answer: As stated before, the changes were made.**

Overall, English language should be significantly improved. For example:
Many words are used improperly (e.g. at line 2: "affection" is not a synonym for "disease")
Many sentences are not clear (e.g. at line 24-25 "among the cognitive domains" does not fit well in the sentence)

**Answer: The text was changed, thank you.**

**Additional Editor Comments:**

Please revise based on two Reviewers comments and also address the following issues.

1. The manuscript is written poorly. Thorough language and grammatical errors should be fixed throughout the manuscript. Compound sentences can be split and made simpler sentences for easier understanding.

**Answer: Thank you for the observation. The manuscript was thoroughly revised.**

2. In the legend to Figure 2, the sentence, ‘The behavior of non-treated SAMP-8 was first compared to SAMR-1 (A and D) and then compared to SAMR-1 (B and E) and SAMP-8 Li+ (C and F). This sentence confuses the readers and requires correction. If the comparisons are made between SAMR-1 untreated vs Li-treated and SAMP-8 untreated vs. Li-treated in the same graph will be easier to understand for each of latency and errors.

**Answer: The legend was rewritten.**

3. In Figure 3, mention what the color codes indicate on the figure for easier understanding. Also, instead of b: p < 0.0001, use some symbols rather than letters to indicate the level of significance.

**Answer: As per the reviewers' suggestion, the figure was changed, including the symbols that indicate the significance of differences.**

4. Western blot images in Figure 4 are too dark, make it lighter to appreciate the differences. It is also very crucial to indicate the position of markers, one below and another above the band.

**Answer: The figure was changed.**

5. In both Figures 5 and 6, data for SAMR-1 plus lithium should be shown for comparison.

**Answer: We don’t have this data, as in previous works, animals with no diseases did not show any difference regarding the behavior or the cellular markers (Nunes et al., 2015). In this way, and concerning the ethical use of laboratory animals, SAMR-1 were not treated with lithium in the present work.**

6. In Figures 6-8, an enlarged version of the brain regions should be shown to reflect whether NeuN-positive neurons and GFAP-positive astrocytes exhibit their characteristic morphology.

**Answer: Unfortunately, we do not have images with larger magnification regarding this experiment.**

7. In figure 9, there is no appreciable difference in Aβ plaques between SAMR-1 and SAMP8 mice. Quantification of Aβ levels should be carried out and shown. It is also important to use Aβ-specific antibody and quantify Aβ levels and plaque density.

**Answer: The image was improved, and the plaques were indicated. Unfortunately, we do not have any further samples of those animals to make an assay like wetern-blot and use Aβ specific antibody. Also, thioflavin S is largely accepted as a specific marker for senile plaques (MacKeigan et al.,** Molecules **2023**, 28(11), 4483; [**https://doi.org/10.3390/molecules28114483**](https://doi.org/10.3390/molecules28114483)).
